# Unsteady Heat and Mass Transfer of Chemically Reacting Micropolar Fluid in a Porous Channel with Hall and Ion Slip Currents

**DOI:** 10.1155/2014/646957

**Published:** 2014-10-29

**Authors:** Odelu Ojjela, N. Naresh Kumar

**Affiliations:** Department of Applied Mathematics, Defence Institute of Advanced Technology (Deemed University), Pune 411025, India

## Abstract

This paper presents an incompressible two-dimensional heat and mass transfer of an electrically conducting micropolar fluid flow in a porous medium between two parallel plates with chemical reaction, Hall and ion slip effects. Let there be periodic injection or suction at the lower and upper plates and the nonuniform temperature and concentration at the plates are varying periodically with time. The flow field equations are reduced to nonlinear ordinary differential equations using similarity transformations and then solved numerically by quasilinearization technique. The profiles of velocity components, microrotation, temperature distribution and concentration are studied for different values of fluid and geometric parameters such as Hartmann number, Hall and ion slip parameters, inverse Darcy parameter, Prandtl number, Schmidt number, and chemical reaction rate and shown in the form of graphs.

## 1. Introduction

The flow and heat transfer of a fluid through porous channels is of great importance in both engineering and science. Applications of these were found in different areas such as oil exploration, geothermal energy extractions, the boundary layer control, extrusion of polymer fluids, solidification of liquid crystals, cooling of a metallic plate in a bath, MHD power generators, and suspension solutions. Many authors investigated the two-dimensional incompressible fluid flow through porous channels theoretically. Terrill and Shrestha [1] considered the laminar incompressible flow of an electrically conducting viscous fluid through a porous channel and gave a solution for a large suction Reynolds number and Hartmann number. Eringen [2, 3] initiated the theory of micropolar fluids and this theory constitutes a subclass of microfluids. Attia [4] considered the problem on MHD flow of a dusty fluid in a circular pipe with Hall and ion slip and obtained a series solution for reduced governing equations. A perturbation solution was obtained for heat and mass transfer in a rotating vertical channel with Hall current by Ahmed and Zueco [5]. Eldabe and Ouaf [6] investigated the problem on MHD flow and heat and mass transfer of a micropolar fluid over a stretching surface with ohmic heating. Magyari et al. [7] have studied Stokes' first problem for micropolar fluid and solved the problem analytically by Laplace transforms and numerically by Valkó-Abate procedure. Bhattacharyya et al. [8] studied the effects of chemical reaction on the boundary layer flow of viscous fluid and a numerical solution obtained by shooting method. The problem of steady incompressible MHD flow of a micropolar fluid between concentric porous cylinders was discussed by Srinivasacharya and Shiferaw [9] who obtained a numerical solution by quasilinearization technique. The steady micropolar fluid flow between two vertical porous parallel plates in the presence of magnetic field was considered by Bhargava et al. [10] and the governing differential equations have been solved using the quasilinearization method. Abdulaziz et al. [11] examined the MHD fully developed natural convection flow of micropolar fluid between parallel vertical plates and the reduced governing nonlinear equations are solved by using HAM method. Das [12] studied the effects of thermal radiation and chemical reaction on incompressible flow of electrically conducting micropolar fluid over an inclined plate. Pal et al. [13] investigated the problem of oscillatory mixed convection-radiation of a micropolar fluid in a rotating system with Hall current and chemical reaction effects and obtained a solution using perturbation method. Rahman and Al-Lawatia [14] examined micropolar fluid flow over a stretching sheet with variable concentration and solved numerically using the shooting method. Bakr [15] considered the steady as well as unsteady MHD micropolar fluid with constant heat source and chemical reaction effect in a rotating frame of reference and solved by using the perturbation method. Patil and Kulkarni [16] studied the free convective flow of a polar fluid in a porous medium in the presence of an internal heat generation with chemical reaction effect. The magnetomicropolar fluid flow and heat and mass transfer in a porous medium with Hall and ion slip effects were discussed by Motsa and Shateyi [17] who obtained a numerical solution using the successive linearization method. Olajuwon [18] studied the heat and mass transfer of an electrically conducting micropolar fluid flow over a horizontal flat plate in the presence of transverse magnetic field with the chemical reaction and Hall effects and the problem is solved by using Runge-Kutta method. Ariman and Cakmak [19] analyzed the three basic viscous flows of micropolar fluid between parallel plates and solved analytically. Modather et al. [20] examined an incompressible MHD flow and heat and mass transfer of a micropolar fluid over a vertical plate in a porous medium. Kim [21] investigated an incompressible micropolar fluid flow past a semi-infinite plate in a porous medium and obtained an analytical solution by perturbation method. Reddy Gorla et al. [22] obtained a numerical solution for the steady boundary layer flow and heat transfer of a micropolar fluid over a flat plate. The MHD flow of viscoelastic fluid over a porous stretching sheet was analyzed by Hymavathi and Shanker [23] and a numerical solution was obtained by using the quasilinearization method. Bhatnagar et al. [24] discussed the steady incompressible laminar flow of viscoelastic fluid through a porous cylindrical annulus and the reduced governing equations are solved numerically using quasilinearization method.

This paper deals with the unsteady two-dimensional incompressible MHD flow and heat transfer of a micropolar fluid in a porous medium between parallel plates with chemical reaction, Hall and ion slip effects. The flow is generated due to periodic suction or injection at the plates and the reduced flow field equations are solved numerically using the quasilinearization technique. The effects of various parameters on velocity components, temperature distribution, microrotation, and concentration are studied and shown graphically.

## 2. Formulation of the Problem

Consider an incompressible electrically conducting micropolar fluid flow through a porous medium between two parallel plates at *y* = 0 and *y* = *h* (Figure 8). The lower and upper plates are subjected to periodic injection and suction of the forms Real (*V*_1_ *e* ^*iΩt*^) and Real (*V*_2_ *e* ^*iΩt*^), respectively. Assume that the temperature and concentration at the lower and upper plates are *T*_1_ *e* ^*iΩt*^, *T*_2_ *e* ^*iΩt*^ and *C*_1_ *e* ^*iΩt*^, *C*_2_ *e* ^*iΩt*^, respectively.

The equations governing the flow and heat and mass transfer in the presence of a magnetic field and in the absence of body forces and body couples are given by Shercliff [25]:

(1) $$\begin{array}{c} \nabla \cdot \bar{q}=0, \end{array}$$

(2) ρ∂q¯∂t+q¯·∇q¯=−gradp+k1curl⁡l¯−μ+k1  ×curl⁡q¯−μ+k1k2q¯+J¯×B¯,

(3) $$\begin{array}{c} \rho j\left[\frac{\partial \bar{l}}{\partial t}+\left(\bar{q}\cdot \nabla \right)\bar{l}\right]=-2k_{1}\bar{l}+k_{1}\mathrm{curl}\bar{q}-\gamma \;\mathrm{curl}\;\mathrm{curl}\;\bar{l}, \end{array}$$

(4) ρc∂T∂t+q¯·∇T=k∇·∇T+2μD:D +k12curl⁡q¯−2l¯2+γ∇l¯:∇l¯ +μ+k1k2q¯2+J¯2σ,

(5) $$\begin{array}{c} \left[\frac{\partial C}{\partial t}+\left(\bar{q}\cdot \nabla \right)C\right]=D_{1}\nabla^{2}C-k_{3}\left(C-C_{1}\right). \end{array}$$

The coefficients *µ*, *k*_1_, *α*, *β*,  *γ* in the above equations are related by the inequalities

(6) $$\begin{array}{c} 2\mu +k_{1}\geq 0,\;k_{1}\geq 0,\;3\alpha +\beta +\gamma \geq 0,\;\gamma \geq \left|\beta \right|. \end{array}$$

Neglecting the displacement currents, the Maxwell equations and the generalized Ohm's law are

(7) $$\begin{array}{c} \nabla \cdot \overset{-}{B}=0,\;\;\nabla \times \overset{-}{B}={µ}^{\text{'}}\overset{-}{J},\;\;\nabla \times \overset{-}{E}=\frac{\partial \bar{B}}{\partial t},\\ \bar{J}=\sigma \left(\bar{E}+\bar{q}\times \bar{B}\right)-\frac{\beta e}{B_{0}}\left(\bar{J}\times \bar{B}\right)+\frac{\beta e\beta i}{B_{0}^{2}}\left(\bar{J}\times \bar{B}\right)\times \bar{B}, \end{array}$$

where $\bar{B}=B_{0}\hat{k}+\bar{b}$, $\bar{b}$ is induced magnetic field, *βe* is the Hall parameter, *βi* is the ion slip parameter, and *µ*′ is magnetic permeability.

For mixed suction case, that is, |*V*_2_ | ≥|*V*_1_|, following Terril and Shreshta [1], we take the velocity, microrotation, temperature distribution, and concentration as

(8) $$\begin{array}{c} \overset{-}{q}=u\hat{i}+v\hat{j},\;\;\overset{-}{l}=N\hat{k}, \end{array}$$

where

(9) u(x,λ,t)=U0a−V2xhf'λeiΩt,vx,λ,t=V2fλeiΩt,Nx,λ,t=1hU0a−V2xhgλeiΩt,Tx,λ,t =T1+μ+k1V2ρhcφ1λ+U0aV2−xh2φ2λeiΩt,Cx,λ,t =C1+nA˙hυG1λ+U0aV2−xh2G2λeiΩt,

where *λ* = *y*/*h*, *U*_0_ is the average entrance velocity, *a* = 1 − (*V*_1_/*V*_2_), and *f*(*λ*), *g*(*λ*), *ϕ*_1_(*λ*), *ϕ*_2_(*λ*), *G*_1_(*λ*), and *G*_2_(*λ*) are functions of *λ* to be determined.

The boundary conditions of the velocity components, microrotation, temperature, and concentration are

(10) $$\begin{array}{c} u\left(x,\lambda ,t\right)=0,\;\;v\left(x,\lambda ,t\right)=V_{1}e^{i\Omega t},\\ N\left(x,\lambda ,t\right)=0,\;\;T\left(x,\lambda ,t\right)=T_{1}e^{i\Omega t},\\ C\left(x,\lambda ,t\right)=C_{1}e^{i\Omega t}\;\text{at}\;\;\lambda =0,\\ u\left(x,\lambda ,t\right)=0,\;\;v\left(x,\lambda ,t\right)=V_{2}e^{i\Omega t},\\ N\left(x,\lambda ,t\right)=0,\;\;T\left(x,\lambda ,t\right)=T_{2}e^{i\Omega t},\\ C\left(x,\lambda ,t\right)=C_{2}e^{i\Omega t}\;\text{at}\;\;\lambda =1. \end{array}$$

Substituting (9) into (2), (3), (4), and (5) and then comparing the real parts on both sides, we get

(11) Reff”'−f'f′′cos⁡ψ =Rg′′+1+Rf'V  −Ha2αe2+βe2αef′′−1+RD−1f′′,J1fg'−f'gcos⁡ψ=−s12g+f′′+g′′,φ1′′+2φ2 +Re Pr⁡Ha21+Rαe2+βe241+Rf'2+s2(1+R)Pr⁡g2      +Ha21+Rαe2+βe2f2+D−1f2−fϕ1'   ×cos⁡ψ=0,φ2′′+Re Pr⁡ ×2f′φ2−fφ2′+f′′21+R+R21+Rf′′+2g2   +Ha21+Rαe2+βe2f′2+D−1f′2f′′21+R ×cos⁡ψ+Re1+Rs2g′2 cos⁡ψ=0,G1′′=−2G2+Kr G1+Sc RefG1′cos⁡ψ,G2′′=Kr G2+Sc Re(fG2′−2f′G2)cos⁡ψ,

where prime denotes the differentiation with respect to *λ* and *αe* = 1 + *βeβi*.

From (9), the dimensionless forms of temperature and concentration are

(12) $$\begin{array}{c} T^{*}=\frac{T-T_{1}e^{i\Omega t}}{\left(T_{2}-T_{1}\right)e^{i\Omega t}}=E\left(\phi_{1}+\zeta^{2}\phi_{2}\right),\\ C^{*}=\frac{C-C_{1}e^{i\Omega t}}{\left(C_{2}-C_{1}\right)e^{i\Omega t}}=\text{Sh}\left(G_{1}+\zeta^{2}G_{2}\right), \end{array}$$

where *E* = (*μ* + *k*_1_)*V*_2_/*ρhc*(*T*_2_ − *T*_1_) is the Eckert number and *ζ* = ((*U*_0_/*aV*_2_) − (*x*/*h*)) is the dimensionless axial variable.

The boundary conditions (10) in terms of *f*, *g*, *ϕ*_1_, *ϕ*_2_, *G*_1_, and *G*_2_ are

(13) $$\begin{array}{c} f\left(0\right)=1-a,\;\;f\left(1\right)=1,\\ f^{\text{'}}\left(0\right)=0,\;\;f^{\text{'}}\left(1\right)=0,\\ g\left(0\right)=0,\;\;g\left(1\right)=0,\\ \phi_{1}\left(0\right)=0,\;\;\phi_{1}\left(1\right)=\frac{1}{E},\\ \phi_{2}\left(0\right)=0,\;\;\phi_{2}\left(1\right)=0,\\ G_{1}\left(0\right)=0,\;\;G_{1}\left(1\right)=\frac{1}{\text{Sh}},\\ G_{2}\left(0\right)=0,\;\;G_{2}\left(1\right)=0. \end{array}$$

## 3. Solution of the Problem

The nonlinear differential equations (11) are converted into the system of first order differential equations by the following substitution:

(14) (f,f',f′′,f”',g,g',ϕ1,ϕ1',ϕ2,ϕ2',G1,G1',G2,G2')=x1,x2,x3,x4x5,x6,x7,x8,x9,x10,x11,x12,x13,x14,dx1dλ=x2,  dx2dλ=x3,  dx3dλ=x4,dx4dλ=11+RHa2αe2+βe2Rex1x4−x2x3cos⁡ψ      −Rs1x3+2x5         +J1x1x6−x2x5cos⁡ψ      +Ha2αe2+βe2αex3+D−1x3,dx5dλ=x6,dx6dλ=s1x3+2x5+J1x1x6−x2x5cos⁡ψ,dx7dλ=x8,dx8dλ=−2x9 −Re Pr⁡41+Rx22+s2Pr⁡1+Rx52      +Ha21+Rαe2+βe2x12      +D−1x12−x1x841+Rx22+s2Pr⁡1+R41+Rcos⁡ψ,dx9dλ=x10,dx10dλ=−Re Pr⁡ ×Ha21+Rαe2+βe2x22+D−1x222x2x9−x1x10+11+Rx32   +R21+Rx32+4x52+4x3x5   +Ha21+Rαe2+βe2x22+D−1x22 ×cos⁡ψ−Re1+Rs2x62cos⁡ψ,dx11dλ=x12,dx12dλ=−2x13+Krx11+ScRex1x12cos⁡ψ,dx13dλ=x14,dx14dλ=Krx13+ScRex1x14−2x2x13cos⁡ψ.

The boundary conditions (13) in terms of *x*_1_, *x*_2_, *x*_3_, *x*_4_, *x*_5_, *x*_6_, *x*_7_, *x*_8_, *x*_9_, *x*_10_, *x*_11_, *x*_12_, *x*_13_, *x*_14_ are

(15) $$\begin{array}{c} x_{1}\left(0\right)=1-a,\;\;x_{2}\left(0\right)=0,\\ x_{5}\left(0\right)=0,\;\;x_{7}\left(0\right)=0,\\ x_{9}\left(0\right)=0,\;\;x_{11}\left(0\right)=0,\;\;x_{13}\left(0\right)=0,\\ x_{1}\left(1\right)=1,\;\;x_{2}\left(1\right)=0,\\ x_{5}\left(1\right)=0,\;\;x_{7}\left(1\right)=\frac{1}{E},\\ x_{9}\left(1\right)=0,\;\;x_{11}\left(1\right)=\frac{1}{\text{Sh}},\;\;x_{13}\left(1\right)=0. \end{array}$$

The system of equations (14) is solved numerically subject to the boundary conditions (15) using the quasilinearization method given by Bellman and Kalaba [26].

Let (*x*_*i*_^*n*^, *i* = 1, 2, … 14) be an approximate current solution and (*x*_*i*_^*n*+1^, *i* = 1, 2, … 14) be an improved solution of (14). Using Taylor's series expansion about the current solution by neglecting the second and higher order derivative terms, the coupled first order system (14) is linearized as

(16) dx1n+1dλ=x2n+1,  dx2n+1dλ=x3n+1,  dx3n+1dλ=x4n+1,dx4n+1dλ=11+R−x2nx5n+1Rex1n+1x4n+x4n+1x1n−x2n+1x3n−x2nx3n+1cos⁡ψ      −Rs1x3n+1+2x5n+1         +J1x1n+1x6n+x1nx6n+1−x2n+1x5n             −x2nx5n+1cos⁡ψs1x3n+1+2x5n+1  −11+RRex1nx4n−x2nx3ncos⁡ψ        −RJ1x1nx6n−x2nx5ncos⁡ψ  +Ha2αe  (1+R)(αe2+βe2)x3n+1+D−1x3n+1,dx5n+1dλ=x6n+1,dx6n+1dλ=s1x3n+1+2x5n+1 +J1x1n+1x6n+x1nx6n+1−x2nx5n+1−x2n+1x5ncos⁡ψ −J1x1nx6n−x2nx5ncos⁡ψ,dx7n+1dλ=x8n+1,dx8n+1dλ=−Re Pr⁡8x2nx2n+11+R+2s2x5nx5n+1Pr⁡(1+R)       +2Ha2x1nx1n+11+Rαe2+βe2+2D−1x1nx1n+1       −x1nx8n+1−x8nx1n+18x2nx2n+11+Rcos⁡ψ +Re Pr⁡4x2nx2n1+R+s2x5nx5nPr⁡⁡1+R+Ha2x1nx1n1+Rαe2+βe2       + D−1x1nx1n−x1nx8n4x2nx2n1+R+s2x5nx5nPr⁡⁡1+R+Ha2x1nx1n1+Rαe2+βe2cos⁡ψ−2x9n+1,dx9n+1dλ=x10n+1,dx10n+1dλ=−Re Pr⁡ ×2Ha2x2nx2n+11+Rαe2+βe22x2nx9n+1+2x2n+1x9n−x1nx10n+1   −x1n+1x10n+2x3nx3n+11+R+R21+R   ×2x3nx3n+1+8x5nx5n+1     +4x3nx5n+1+4x3n+1x5n   +2Ha2x2nx2n+11+Rαe2+βe2+2D−1x2n+1x2n2Ha2x2nx2n+11+Rαe2+βe2cos⁡ψ −2Re1+Rs2x6nx6n+1cos⁡ψ +Re Pr⁡ ×2x2nx9n−x1nx10n+x3nx3n1+R+R21+R   ×x3nx3n+4x5nx5n+4x3nx5n   +Ha2x2nx2n1+Rαe2+βe2+D−1x2nx2ncos⁡ψ +Re1+Rs2x6nx6ncos⁡ψ,dx11n+1dλ=x12n+1,dx12n+1dλ=−2x13n+1+Krx11n+1 +ScRex1n+1x12n+x1nx12n+1−x1nx12ncos⁡ψ,dx13n+1dλ=x14n+1,dx14n+1dλ=Krx13n+1+ScRe ×x1n+1x14n+x1nx14n+1−2x2n+1x13n   −2x2nx13n+1−x1nx14n+2x2nx13ncos⁡ψ.

To solve for (*x*_*i*_^*n*+1^, *i* = 1, 2 … 14), the solution to seven separate initial value problems, denoted by *x*_*i*_^*h*1^(*λ*), *x*_*i*_^*h*2^(*λ*), *x*_*i*_^*h*3^(*λ*), *x*_*i*_^*h*4^(*λ*), *x*_*i*_^*h*5^(*λ*), *x*_*i*_^*h*6^(*λ*), *x*_*i*_^*h*7^(*λ*) (which are the solutions of the homogeneous system corresponding to (16), and *x*_*i*_^*p*1^(*λ*) (which is the particular solution of (16), with the following initial conditions, is obtained by using the 4th order Runge-Kutta method:

(17) x3h1(0)=1, xih1(0)=0 for  i≠3,x4h2(0)=1, xih2(0)=0 for  i≠4,x6h3(0)=1, xih3(0)=0 for  i≠6,x8h4(0)=1, xih4(0)=0 for  i≠8,x10h5(0)=1, xih5(0)=0 for  i≠10,x12h6(0)=1, xih6(0)=0 for  i≠12,x14h7(0)=1, xih7(0)=0 for  i≠14,x1p1(0)=1−a,x2p1(0)=x3p1(0)=x4p1(0)=x5p1(0)=0,x6p10=x7p1(0)=x8p1(0)=x9p1(0)=x10p1(0)=x11p1(0)=x12p1(0)=x13p1(0)=x14p1(0)=0.

By using the principle of superposition, the general solution can be written as

(18) xin+1λ=C1xih1(λ)+C2xih2(λ)+C3xih3(λ)+C4xih4λ+C5xih5λ+C6xih6λ+C7xih7λ+xip1λ,

where *C*_1_, *C*_2_, *C*_3_, *C*_4_, *C*_5_, *C*_6_, and  *C*_7_ are the unknown constants and are determined by considering the boundary conditions at *λ* = 1. This solution (*x*_*i*_^*n*+1^, *i* = 1, 2, … 14) is then compared with solution at the previous step (*x*_*i*_^*n*^, *i* = 1, 2, … 14) and further iteration is performed if the convergence has not been achieved.

## 4. Results and Discussions

To understand the flow characteristics in a better way, the numerical results of the axial velocity *u*, radial velocity *v*, microrotation *N*, temperature distribution *T*, and concentration *C* are calculated correct to six places of decimal for various values of nondimensional chemical reaction parameter Kr, ion slip parameter *βi*, Hall parameter *βe*, inverse Darcy parameter *D* ^−1^, Hartmann number Ha, Prandtl number *Pr*⁡, and Schmidt number Sc in the domain [0, 1].

**Figure 1:**
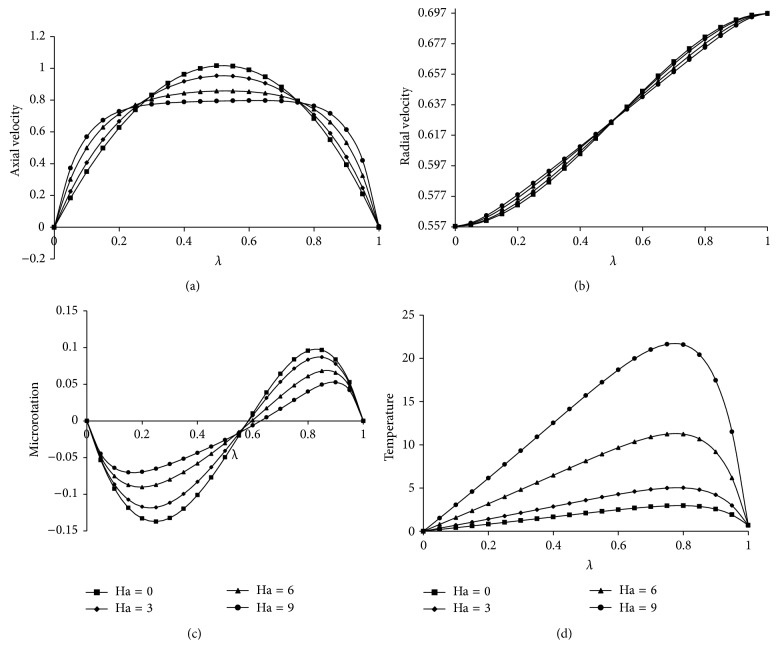
Effect of Ha on (a) axial velocity, (b) radial velocity, (c) microrotation and (d) temperature. For Kr = 20, *Re* = 2, *a* = 0.2, *J*_1_ = 2, *βe* = 0.5, *βi* = 0.5, Sc = 0.22, *Pr*⁡ = 7, *R* = 2, *ψ* = 0.8, *s*_1_ = 2, *s*_2_ = 2, *D* ^−1^ = 2.

**Figure 2:**
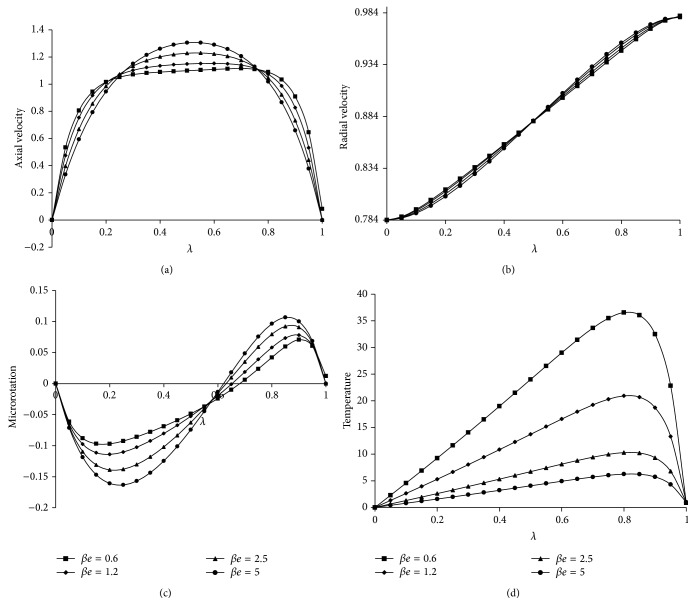
Effect of *βe* on (a) axial velocity, (b) radial velocity, (c) microrotation and (d) temperature. For Kr = 2, *Re* = 2, a = 0.2, *J*_1_ = 2, *βi* = 0.5, Sc = 0.8, *Pr*⁡ = 7, *R* = 2, *ψ* = 0.2, *s*_1_ = 2, *s*_2_ = 2, Ha = 10, *D* ^−1^ = 2.

**Figure 3:**
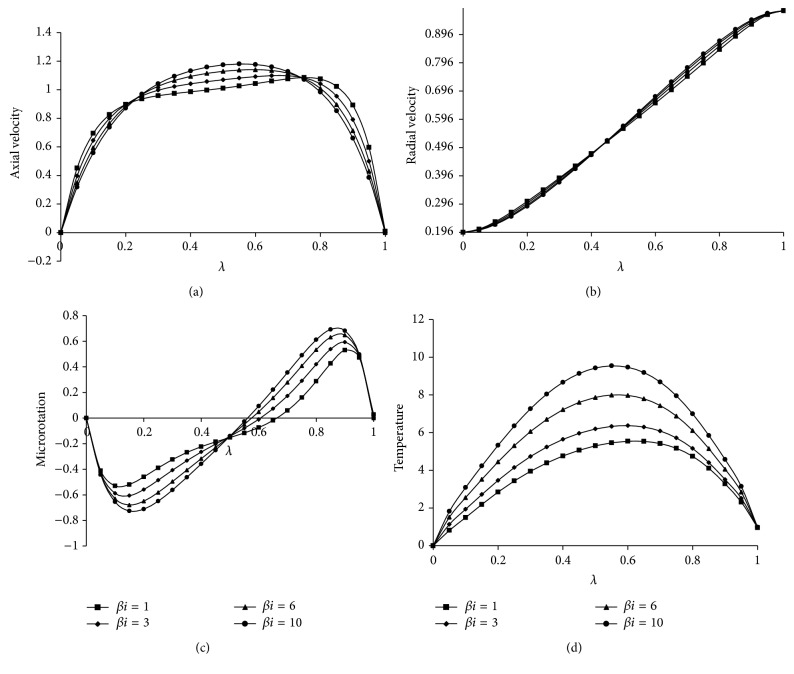
Effect of *βi* on (a) axial velocity, (b) radial velocity, (c) microrotation and (d) temperature. For Kr = 10, *Re* = 2, *a* = 0.2, *J*_1_ = 2, *βe* = 0.5, Sc = 0.22, *Pr*⁡ = 7, *R* = 2, *ψ* = 0.8, *s*_1_ = 2, *s*_2_ = 2, Ha = 5, *D* ^−1^ = 2.

**Figure 4:**
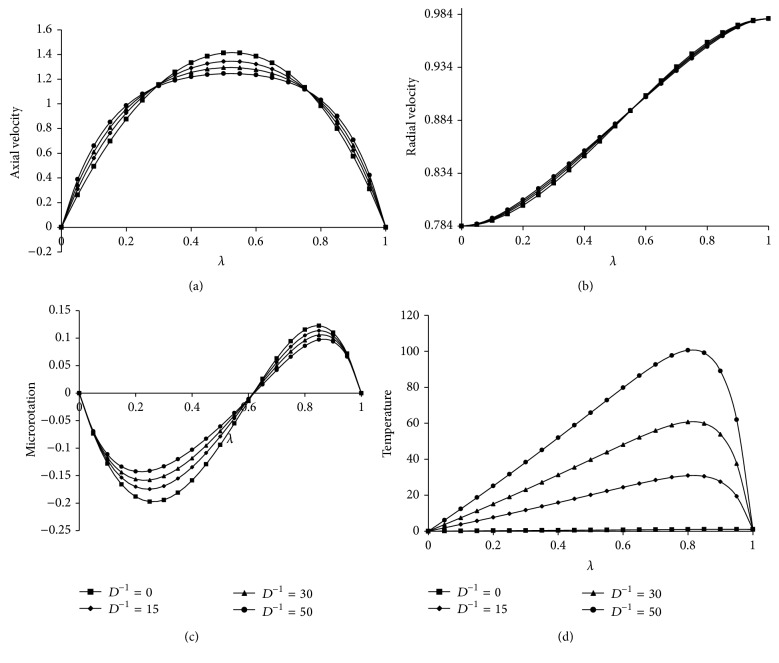
Effect of *D* ^−1^ on (a) axial velocity, (b) radial velocity, (c) microrotation and (d) temperature. For Kr = 3, *Re* = 2, *a* = 0.2, *J*_1_ = 2, *βe* = 0.5, *βi* = 3, Sc = 0.8, *Pr*⁡ = 7, *R* = 2, *ψ* = 0.2, *s*_1_ = 2, *s*_2_ = 2, Ha = 2.

**Figure 5:**
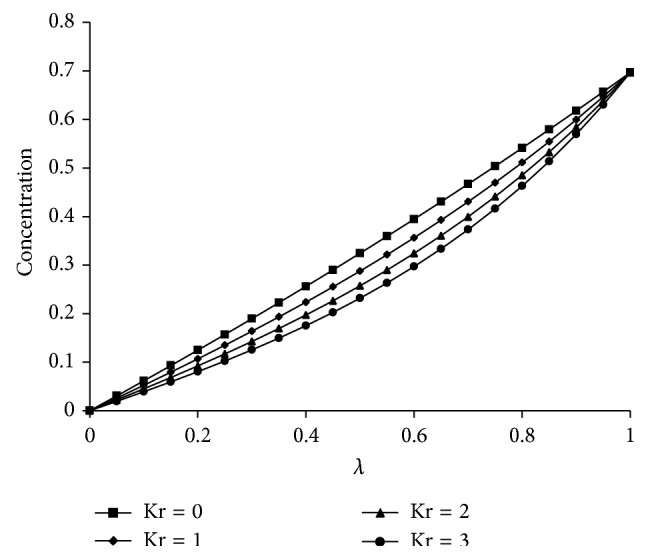
Effect of “Kr” on concentration. For *Re* = 2, *a* = 0.2, *J*_1_ = 2,  *βe* = 0.5, *βi* = 3, Sc = 0.22, *Pr*⁡ = 7, *R* = 2, *ψ* = 0.8, *s*_1_ = 2, *s*_2_ = 2, Ha = 5, and *D* ^−1^ = 50.

**Figure 6:**
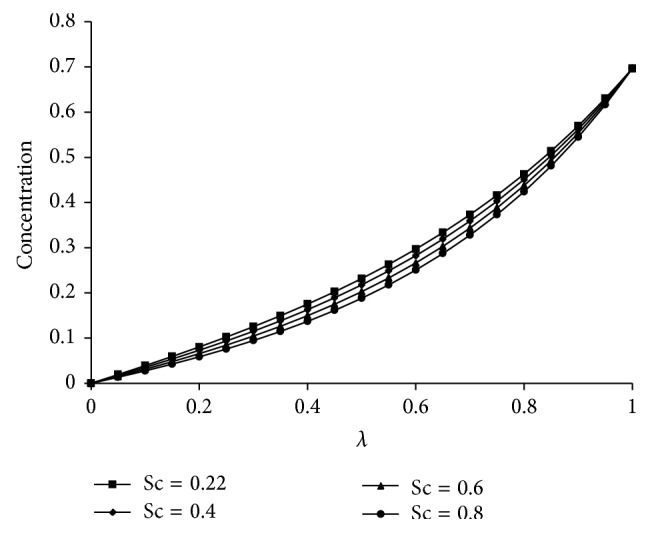
Effect of Sc on concentration. For Kr = 3, *Re* = 2, *a* = 0.2, *J*_1_ = 2, *βe* = 0.5, *βi* = 3, *Pr*⁡ = 7, *R* = 2, *ψ* = 0.8, *s*_1_ = 2, *s*_2_ = 2, Ha = 5, and *D* ^−1^ = 50.

**Figure 7:**
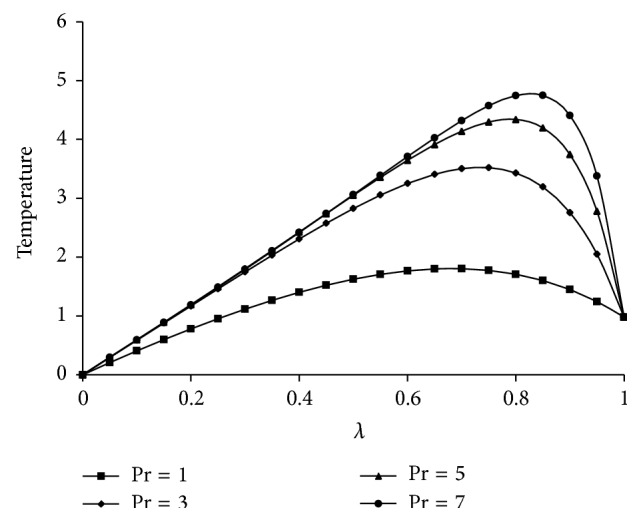
Effect of Pr on temperature. For Kr = 2, *Re* = 2, *a* = 0.2, *J*_1_ = 2, *βe* = 2, *βi* = 1, Sc = 0.8, *R* = 2, *ψ* = 0.2, *s*_1_ = 2, *s*_2_ = 2, Ha = 2, and *D* ^−1^ = 2.

**Figure 8:**
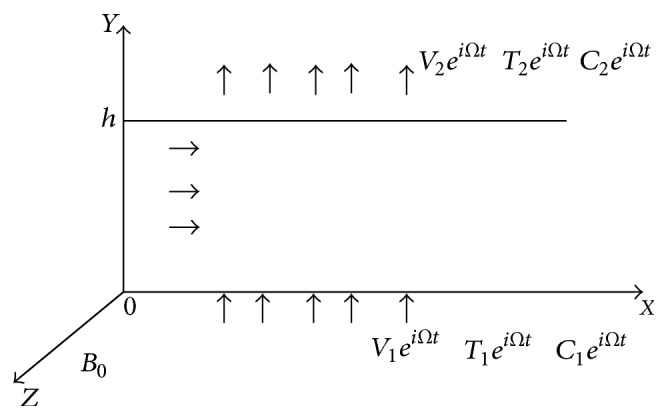
The schematic diagram of fluid flow between two porous parallel plates.

The effect of Ha on velocity components, microrotation, and temperature is presented in Figures 1(a) to 1(d). It is observed that as Ha increases the axial velocity also increases towards the center of the plates and then decreases because of the Lorentz force which tends to resist the flow and the radial velocity, microrotation, and temperature distribution are increasing from the lower plate to the upper plate. Figures 2(a) to 2(d) give the effect of *βe* on velocity components, microrotation, and temperature distribution. It can be analyzed that as *βe* increases the axial velocity also increases in the center of the channel, whereas the temperature decreases towards the upper plate. However, the radial velocity and microrotation are decreasing up to the center of the channel and then increase. This is because of the fact that the *βe* decreases the resistive force imposed by magnetic field. The effect of *βi* on velocity components, microrotation, and temperature distribution has been presented in Figures 3(a) to 3(d), respectively, and these profiles follow the same trend of *βe*. The effect of *D* ^−1^ on velocity components, microrotation, and temperature distribution is shown in Figures 4(a) to 4(d). It can be observed that as *D* ^−1^ increases the radial velocity and microrotation are increasing up to the center of the channel and then decrease towards the upper plate and the axial velocity gives the maximum effect at the center of the plates. However, the temperature increases from lower plate to upper plate. The effects of Kr and Sc on concentration are shown in Figures 5 and 6, respectively. From Figure 5 it is understood that as Kr increases the concentration decreases because the chemical reaction increases the rate of interfacial mass transfer. It is depicted from Figure 6 that the concentration is decreasing as Sc increases and this causes the concentration buoyancy effect.  Figure 7 shows the effect of Pr on temperature. It is noticed that as Pr increases the temperature distribution also increases towards the upper plate and this is due to the fact that as Pr increases the thickness of the boundary layer decreases and this gives the larger temperature values.

## 5. Conclusions

In the present paper, the effects of chemical reaction and Hall and ion slip on unsteady two-dimensional laminar flow of an electrically conducting micropolar fluid through a porous medium between parallel plates with heat and mass transfer are considered. The reduced nonlinear ordinary differential equations are solved by using the quasilinearization method. The results are presented in the form of graphs for various values of fluid and geometric parameters and from these the following is concluded.

- The chemical reaction rate reduces the concentration while the Prandtl number enhances the temperature.
- The effects of Hall parameter and ion slip parameter on velocity components, microrotation, and temperature are similar.
- The Hartmann number and inverse Darcy's parameter have the same result for velocity components, microrotation, and temperature.

## Nomenclature

*h*: Distance between parallel plates
*V*_2_: Suction velocity
*V*_1_: Injection velocity
*a*: Injection suction ratio, 1 − (*V*_1_/*V*_2_)
*p*: Fluid pressure
$\bar{q}$: Velocity vector
*c*: Specific heat at constant temperature
$\bar{l}$: Microrotation vector
*N*: Microrotation component
*E*: Eckert number, (*μ* + *k*_1_)*V*_2_/*ρhc*(*T*_2_ − *T*_1_)
*k*: Thermal conductivity
*k*_1_: Viscosity parameter
*k*_2_: Permeability parameter
*u*: Velocity component in *x*-direction
*v*: Velocity component in *y*-direction
*U*_0_: Average entrance velocity
Pr: Prandtl number, *μc*/*k*
Re: Suction Reynolds number, *ρV*_2_ *h*/*μ*
*j*: Gyration parameter
$\bar{J}$: Current density
*J*_1_: Nondimensional gyration parameter, *ρjhV*_2_/*γ*
$\bar{B}$: Total magnetic field
$\bar{b}$: Induced magnetic field
*B*_0_: Magnetic flux density
*D*: Rate of deformation tensor
$\bar{E}$: Electric field
Ha: Hartmann number, $B_{0}h\sqrt{\sigma /\mu }$
*D*^−1^: Inverse Darcy parameter, *h* ^2^/*k*_2_
*R*: Nondimensional viscosity parameter, *k*_1_/*μ*
*s*_1_: Nondimensional micropolar parameter, *k*_1_ *h* ^2^/*γ*
*s*_2_: Nondimensional micropolar parameter, *γc*/*h* ^2^ *k*
*T*: Temperature
*T*_1_: Temperature at the lower plate
*T*_2_: Temperature at the upper plate
*T*^*^: Dimensionless temperature, (*T* − *T*_1_ *e* ^*iΩt*^)/(*T*_2_ − *T*_1_)*e* ^*iΩt*^
*C*^*^: Dimensionless concentration, (*C* − *C*_1_ *e* ^*iΩt*^)/(*C*_2_ − *C*_1_)*e* ^*iΩt*^
*C*: Concentration
*D*_1_: Mass diffusivity
*k*_3_: Chemical reaction rate
Kr: Nondimensional chemical reaction parameter, *k*_3_ *h* ^2^/*D*_1_
Sc: Modified Schmidt number, *υ*/*D*_1_.

### Greek Letters

*λ*: Dimensionless *y* coordinate, *y*/*h*
*γ*: Gyroviscosity parameter
*ζ*: Dimensionless((*U*_0_/*aV*_2_) − (*x*/*h*))
*ρ*: Fluid density
*μ*: Fluid viscosity
*μ*′: Magnetic permeability
*σ*: Electric conductivity
*ψ*: Nondimensional frequency parameter, *Ωt*
*βi*: Ion slip parameter
*βe*: Hall parameter
*αe*: Hall and ion slip parameters, 1 + *βiβe*.
